# Seroprevalence of Hepatitis E Virus Infection, Rural Southern People’s Republic of China

**DOI:** 10.3201/eid1211.060332

**Published:** 2006-11

**Authors:** Rong-Cheng Li, Sheng-Xiang Ge, Yan-Ping Li, Ying-Jie Zheng, Yi Nong, Qing-Shun Guo, Jun Zhang, Mun-Hon Ng, Ning-Shao Xia

**Affiliations:** * Guangxi Center for Disease Control and Prevention, Nanning, People's Republic of China;; † Xiamen University, Xiamen, People's Republic of China

**Keywords:** hepatitis E, seroepidemiology, endemics, swine, zoonosis, research

## Abstract

Genotype 4 hepatitis E virus (HEV) is the dominant cause of hepatitis E in the People's Republic of China; swine are the principal reservoir. Our study was conducted in 8 rural communities of southern China, where families keep pigs near their homes. Phylogenetic analysis showed that 23 of 24 concurrent virus isolates from this region are genotype 4 strains. Among the study populations, immunoglobulin G anti-HEV seroprevalence accumulated with age at ≈1% per year for persons >60 years of age. After age 30 years, seroprevalence increased at higher rates for male than for female study participants. The overall seroprevalence was 43% (range 25%–66%) among the communities. Infection rates were higher for participants between 25 and 29 years of age. The results suggest that HEV infection probably has been endemic in southern China for <60 years, with swine being the principal reservoir of human HEV infection in recent years.

## Abstract 2

HEV infection is thought to have been endemic in southern China for >60 years; swine are now the main source of human infection.

Hepatitis E virus (HEV), a member of the *Hepevirus* genus, is an RNA-positive strand virus that resembles calicivirus both morphologically and in organization of its 7.5-kb genome (*1*). The virus was first identified as the cause of extended waterborne outbreaks of hepatitis, with significant deaths among pregnant women (*2*). Widely distributed in nature, the virus is detected in swine and other animals—both domestic and wild (*3**–**5*). Based on phylogenetic analysis, the virus isolates can be separated into 4 major groups, genotypes 1–4 (*6*). While viruses of genotypes 1 and 2 are isolated exclusively from humans, those from genotypes 3 and 4 have also been isolated from swine and other animals (*7*). Genotype 1 strains are mainly distributed in Asia and the Middle East (*8**,**9*), where they frequently cause waterborne outbreaks of hepatitis (*10**,**11*). Genotype 2 virus was first detected in Mexico (*12*). Genotype 3 strains are widely distributed; they have been isolated from swine in North America (*13*), South America (*14*), Europe (*15*), Oceania (*16*), and Asia (*17*) and, in some of these areas, from rare, indigenous human cases of hepatitis E (*18**,**19*). Genotype 3 virus isolated from wild pigs and deer in Japan was recently found to be the cause of an outbreak of foodborne infection (*20**,**21*). Genotype 4 is largely restricted to Asia. The virus was detected in archival swine serum samples in India collected in 1985 (*22*) and since then has been detected in swine in Taiwan (*23*), mainland People's Republic of China (*5*), Indonesia (*24*), India (*22*), and Japan (*25*). Genotype 4 virus causes sporadic cases and is associated with foodborne infection but has not been generally associated with waterborne outbreaks. The different HEV genotypes are indistinguishable serologically (*26*), however, and studies in primates show cross-protection among the 4 genotypes (*27**,**28*).

HEV was first detected in People's Republic of China during an extended outbreak in Xinjiang Province in 1986 (*10*). The causal agent was a strain of genotype 1 virus, which persisted as the dominant genotype in China until 2000, when genotype 4 emerged as the dominant genotype (*29**,**30*). A recent study conducted in 2 swine farming districts of eastern China showed that genotype 4 virus freely circulates among swine and humans: an estimated 9% of swine and 0.3% of humans have asymptomatic infection (*31*). In seroepidemiologic studies conducted concurrently by these authors, the risk for human HEV infection was associated with occupational contact with swine and swine sewage, making the animals a principal reservoir for human infection.

We describe a seroepidemiologic study of HEV infection in 8 rural communities of southern China and a phylogenetic analysis of the virus circulating in the region. The results showed that HEV infection is endemic in the region and probably has been for at least 60 years. The prevalent virus population is genetically diverse, although dominated by genetically diverse genotype 4 virus.

## Materials and Methods

### Study Participants

We conducted a cross-sectional and a follow-up seroepidemiologic study of HEV infection in 2003 and 2004 in conjunction with annual health examinations conducted among residents of rural communities in Guangxi Province (GX) in southern China. These exams involve routine clinical examination, routine biochemical testing, and determination of hepatitis A virus (HAV) and hepatitis B virus (HBV) serologic status. Participants were enrolled in the study after providing informed consent; parental consent was obtained for participants <16 years of age. A questionnaire was used to record demographic data, education level, employment, source of water supply, sanitation practices, and household contact with pigs and poultry. Ethical approval for the study was obtained from the Guangxi Institutional Review Board.

### Immunoglobulin G (IgG) anti-HEV Assay

An aliquot of serum was obtained from the samples taken for routine biochemical testing and HAV and HBV status and made available for the present study. Serum samples were tested for IgG anti-HEV by using a commercial ELISA (Wan Tai Pharmaceutical Co., Beijing, China), produced with a recombinant peptide corresponding to amino-acid residues 396 to 606 of the major structural protein specified by open reading frame 2 (ORF2) of the HEV genome (*32*). Serum samples were diluted 1:10, and tested according to manufacturer's instruction. A positive reaction was indicated when the signal:cut-off (S:CO) exceeded 1.5.

### Detection of HEV RNA

For the virologic study, serum samples were taken from 24 patients admitted to local hospitals in 2003 and 2004 with serologically diagnosed hepatitis E. Total RNA was extracted from 250 μL of sample with Trizol (Invitrogen). Reverse-transcription polymerase (RT)-PCR was performed as described previously (*33**,**34*). Briefly, a 150-nt segment of ORF2, was amplified with primers E1 (5´-CTGTTTAA[C/T]CTTGCTGA CAC-3´,6,260–6,279) and E5 (5´-(A/T)GA[A/G]AGCCAAAGCACATC-3´, nt 6,568–6,551) in the first round of PCR and primers E2 (5´-GACAGAATTGATTTCGTCG-3´, nt 6,298–6,316) and E4 (5´-TG[C/T]TG GTT[A/G]TC[A/G]TAATCCTG-3´, nt 6,486–6,467) in the second round. PCR cycling conditions for both rounds consisted of 35 cycles of denaturation at 94°C for 30 s, annealing at 53°C for 30 s, and extension at 72°C for 40 s. PCR products were purified and sequenced in a forward and reverse direction by using an automatic nucleotide sequencer (ABI model 3730 sequencer, Applied Biosystems, Foster City, CA, USA).

### Phylogenetic Analysis

A 150-nt ORF2 segment was amplified from 24 virus isolates from patients with serologically diagnosed acute hepatitis E who were admitted to local hospitals during the period of study. The nucleotide sequence of the amplified products and that of prototypes of different genotypes of HEV strains were aligned by using the MEGA 3.0 software (version 3.0,http://www.megasoftware.net). Genomic sequences of prototype HEV strains (Burma1, M73218; Burma2, D10330; CN-Xinjiang, D110920; CN-genotype4, AJ272108; India1, X98292; Pakistan, M80581; Mexico, M74506; U.S., AF060668) were obtained from GenBank. Phylogenetic trees were generated by the minimum evolution method and the interior branch method; 1,000 resamplings of the data were used to calculate percentage of the branches obtained. The identity between the nucleotide sequences was calculated by using the program MegAlign (DNAstar package version 5.03; Lasergene, DNAstar Inc., Madison, WI, USA).

### Statistical Methods

Seroprevalence was standardized for age and sex according to China's national census of 2000. Multivariate unconditional logistic regression analysis was performed with SAS (Version 8.2; SAS Institute Inc., Cary, NC, USA) and was used to identify independent determinants of IgG anti-HEV prevalence and to estimate the level of the associated risk (*35*).

## Results

Serum samples were taken in 2003 from 7,284 participants; a second sample was taken 12 months later from a subpopulation of 3,431 of these persons (Table 1). The study participants were recruited from 8 rural communities situated 50–500 km of one another in southern China. The water supply for this area comes from wells, rivers, and streams. Tap water and sewage treatment are not generally available. The study population was of similar socioeconomic and cultural backgrounds, and, in general, participants had been residing in their respective communities for most of their lives. Farming is the major source of income; most families rear swine and other domestic animals for their own consumption and for sale to supplement family incomes. Within these communities, men commonly migrate to urban centers to seek employment; hence, female participants predominate in the study population.

**Table 1:** Study participants*

Community	2003					2004				
	n	Age (y)			Sex ratio (M:F)†	n	Age (y)			Sex ratio (M:F)
		Mean	SD	Range			Mean	SD	Range	
Linyun	1,047	24.1	15.4	1–74	0.90	738	22.9	15.4	1–72	0.96
Xin'an	1,037	31.3	17.9	1–87	0.85	533	31.8	18.4	1–87	0.77
Guilin	542	35.3	15.0	0.9–80	1.14	340	37.3	14.1	1–80	1.06
Luochen	981	32.5	19.0	1–85	0.73	455	34.2	19.7	1–79	0.72
Tiendan	377	35.1	16.4	1–70	0.98	166	37.2	16.4	2–70	0.73
Binyan	1,106	28.9	18.2	1–78	0.92	657	28.5	19.1	1–78	0.84
Linshan	1,230	27.4	20.5	0.4–87	0.94	542	26.2	21.6	0.5–80	0.97
Liuzhen†	964	44.6	16.8	0.1–79	0.88	–	–	–	–	–
Total	7,284	31.7	18.8	0.1–87	0.89	3,431	29.5	18.8	0.5–87	0.87
M	3,440	30.2	19.4	0.4–85	–	1,597	27.9	19.3	0.5–80	–
F	3,844	33.0	18.8	0.1–87	–	1,834	30.9	18.3	1–87	–

*Serum samples were taken in 2003 from 7,284 persons residing in 8 communities of Guangxi Province. Second samples were taken 12 mo later from a subpopulation of 3,431 study participants residing in 7 of these communities. *SD, standard deviation;, M = male; F = female.†Dash indicates "not done" in reference to Liuzhen and "not applicable" in reference to sex.

Table 2 shows the IgG anti-HEV status of the 7,284 study participants from these 8 rural communities in 2003. The age and sex-standardized IgG anti-HEV seroprevalence is 43.5% for the general study population and 25.2%–66.1% for the 8 communities. The age-standardized seroprevalence is 47.1% for male participants and 39.7% for female ones. Multivariate unconditional logistic regression analysis identified age, sex, and community to be independent determinants for IgG anti-HEV seroprevalence, but not other factors such as income, employment, education, and source of water supply. Because rearing pigs and chickens adjacent to one's home is common, most families reside in close proximity to domestic animals whether or not they keep them themselves. Consequently, assessing the risk for infection attributable to this practice was not possible.

**Table 2:** IgG anti–hepatitis E virus (HEV) seroprevalence in rural communities, southern China, 2003

Communities	Participants	IgG anti-HEV seroprevalence (%)*	
		Observed	Standardized
Linyun	1,047	58.4	66.1
Xin'an	1,037	60.4	59.4
Guilin	542	57.8	49.4
Luochen	981	42.2	42.6
Tiendan	377	45.6	38.8
Binyan	1,106	31.1	36.2
Linshan	1,230	21.0	25.2
Liuzhen	964	43.1	30.4
Total	7,284	43.3	43.5
Male	3,440	45.8	47.1
Female	3,844	41.0	39.7

*Seroprevalence standardized according to People's Republic of China national census of 2000; IgG, immunoglobulin G.

**Figure 1:**
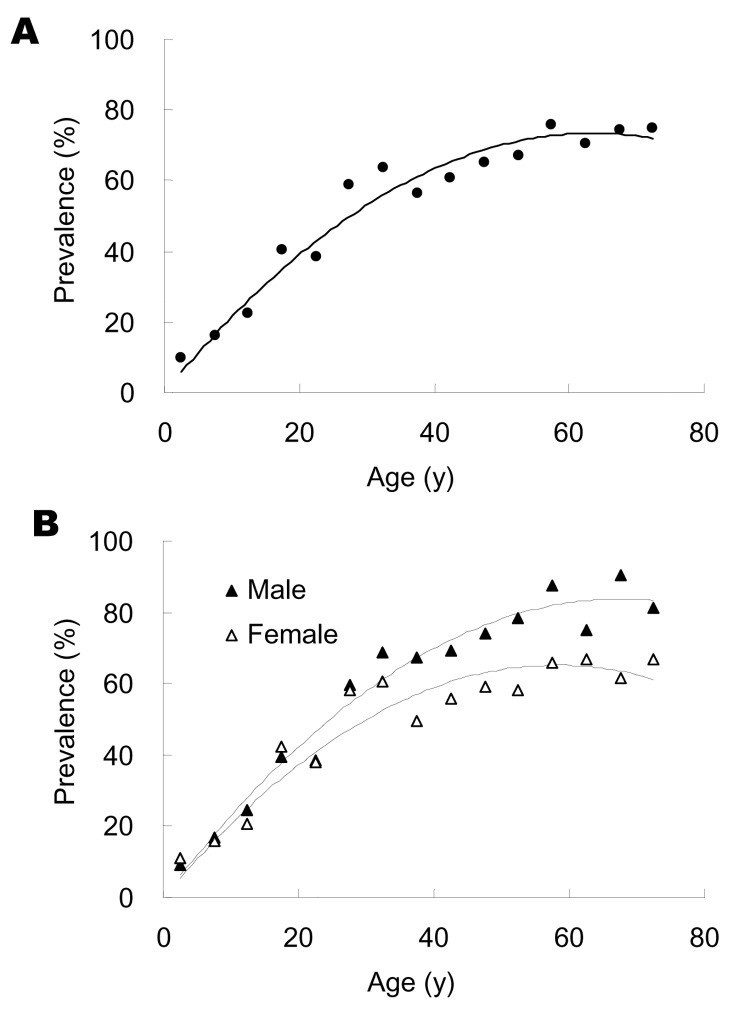
Age specific anti–hepatitis E virus (HEV) seropositive rates in a study population. Age-specific IgG anti-HEV seropositive rates for (A) both sexes or (B) either sex separately (black triangle for male study participant, open triangle for female) were determined for every 5 years from 0 to 69 years of age and for older participants, using samples taken in 2003 from 7,284 persons.

On the basis of results obtained with the samples taken in 2003, the age-specific IgG anti-HEV seropositive rate was calculated in 5-year increments up to 69 years of age and summarily for older participants. The IgG anti-HEV seropositive rate of the general population (Figure 1A) accumulated with age for both male and female participants (Figure 1B). The seropositive rate of the general study population increased at a relatively constant rate of ≈1% per year up to ≈60 years of age, and then remained essentially stable. IgG anti-HEV seropositive rate increased at a similar rate for male and female participants <30 years of age then at higher rate for male participants 30–59 years of age (Figure 1B). Multivariate unconditional logistic regression analysis estimated that IgG anti-seropositive rates were similar for male and female participants up to age 30 years (odds ration [OR] = 0.9, 95% confidence intervals [CI] = 0.7–1.1) but were ≈2-fold higher for male participants >30 years of age (OR = 2.1, 95% CI = 1.7–2.6).

Changes in IgG anti-HEV serologic status occurring in 2004 were determined by using a second sample taken 12 months later from a subpopulation of 3,431 participants (Table 3) from 7 of the 8 communities. The results showed that 4.3% of seronegative participants seroconverted to positive, and 1.4% of seropositive participants underwent negative seroconversion. The overall seropositive rate of the subpopulation increased from 46.2% in 2003 to 49.1% in 2004. The positive seroconversion rate was higher for male participants, but the difference was not statistically significant (p>0.05). One community (Linyun) evidently experienced an undetected outbreak of HEV infection between 2003 and 2004, since its positive seroconversion rate of 17.9% was significantly higher than the positive seroconversion rates of the other communities (p<0.01). In those communities, positive seroconversion rates ranged from 0.7% to 4.2%, but the differences between them were not statistically significant (p>0.05). The negative seroconversion rates were 0%–3.03% among the different communities, also not statistically significant (p > 0.05).

**Table 3:** Changes in IgG anti-hepatitis E virus (HEV) status in rural communities of southern China, 2003–2004

Communities	Participants	IgG anti-HEV seroprevalence (%)		PC* % (n)	NC* % (n)
		2003	2004		
Linyun	738	62.2	78.2	17.9 (50)*	2.0 (9)
Xin'an	533	63.6	65	3.1 (6)	0.3 (1)
Guilin	340	58.2	59.4	4.2 (6)	3.0 (6)
Luochen	455	44.4	44.9	2.0 (5)	1.5 (3)
Tiendan	166	48.2	46.9	1.2 (1)	2.5 (2)
Binyan	657	30.6	31.8	1.8 (8)	0.5 (1)
Linshan	542	19.4	20.1	0.7 (3)	0.0 (0)
Liuzhen†	–	–	–	–	–
Total	3,431	46.2	49.1	4.3 (79)	1.4 (22)
Male	1,597	48.5	52.1	5.0 (41)	1.4 (11)
Female	1,834	44.1	46.5	3.7 (38)	1.4 (11)

*Changes in serologic status were indicated by changes in IgG anti-HEV seroprevalence observed in 2003 and 2004, percent and number (n) of seronegative participants undergoing positive seroconversion (PC), and that of seropositive persons undergoing negative seroconversion (NC). Linyun has significantly higher PC (p<0.01). †–, not done.

**Figure 2:**
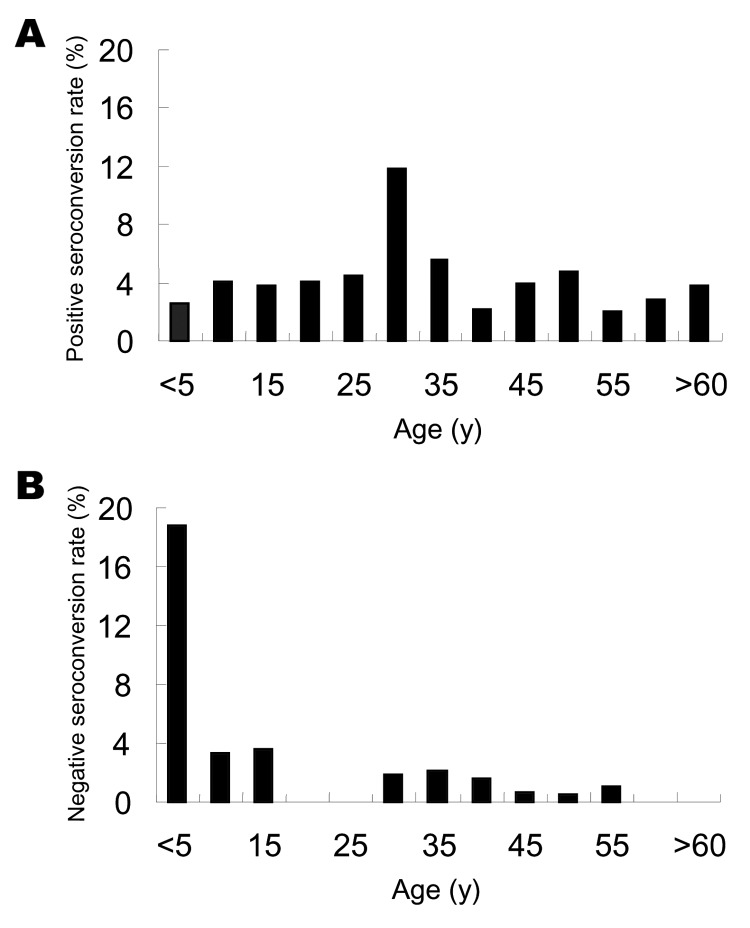
Changes in immunoglobulin G (IgG) anti-hepatitis E virus (HEV) serologic status, 2003–2004. Age-specific IgG anti-HEV–positive seroconversion (A) and age-specific IgG anti-HEV–negative seroconversion (B), determined for every 5 years of age from 0 to 59 years of age and in older participants, using samples taken 12 months apart from a subpopulation of 3,431 persons.

Age-specific IgG anti-HEV positive seroconversion rates and negative seroconversion rates were calculated in increments of 5 years up to 59 years of age (for 2003, it was 69 years) and then summarized for the older participants. Figure 2A shows that positive seroconversion occurred at all ages. Highest rates occurred among persons in the 25- to 29-year age group (11.8% per year) and ranged from 2.1% to 5.6% per year for the other age groups. On the basis of multivariate unconditional logistic regression analysis, the risk for infection was ≈3-fold higher for persons 25–29 years of age than for those in the other age groups (OR = 3.2, 95% CI = 1.7–5.8).

The overall rate of negative seroconversion was 1.4%. For the 0–4 year age group, this rate was 18.8%, which is ≈15 times higher that of the older age groups (OR = 14.9, 95% CI = 4.9–45.4); however, this rate likely reflects the loss of maternal antibodies. Among the older age groups, negative seroconversion occurred at similar rates of 0 to 3.6% per year.

**Figure 3:**
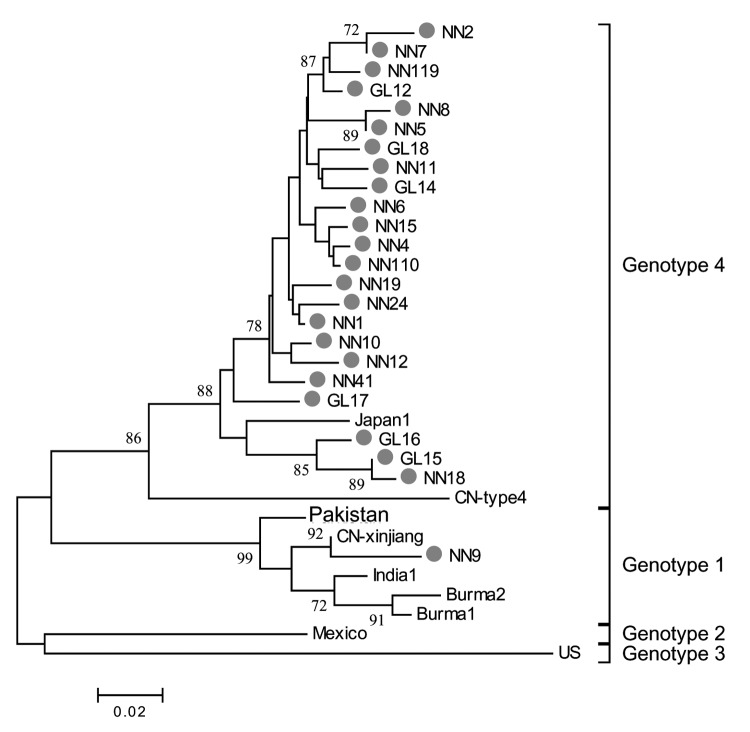
Phylogenetic analysis of hepatitis E virus (HEV) isolates. HEV isolates from patients with serologically diagnosed hepatitis E cases admitted in 2003 and 2004 to local hospitals are represented by closed circles. Prototype strains of indicated genotypes are designated according to site of isolation. Numbers on the branches represent (percent) reproduced values calculated from 1,000 resamplings of the data. The bar represents a genetic distance of 0.02-nt substitution per position.

All HEV infections observed among the study participants were asymptomatic; no cases of overt hepatitis E were observed. To study HEV prevalence in the communities during our study period, we obtained 24 HEV strains isolated from persons with serologically diagnosed hepatitis E who were admitted to hospitals in Guangxi between 2003 and 2004. Figure 3 shows the phylogenetic tree produced with the alignments of a 150-nt ORF2 sequence. All 24 isolates had distinct nucleotide sequences. One isolate was a genotype 1 virus, which is more closely related to prototype Chinese than to Burmese, Indian, or Pakistani genotype 1 strains. The other 23 isolates were genotype 4 virus related to the Japanese prototype genotype 4 strain. Genotype 4 isolates are genetically diverse, and in our study genetic identity between pairs of isolates ranged from 88.7% to 99.3%, with a mean value of 95.4%.

## Discussion

We undertook a cross-sectional seroepidemiologic study in 2003 to determine the extent of HEV infection occurring in 8 rural communities in southern China. To determine new infections, we obtained a second serum sample from a subgroup of the 2003 study population after 12 months. Genetic analysis was performed on 24 strains of HEV isolated from patients with serologically diagnosed hepatitis E who were admitted to hospitals in the region during the study period.

The IgG anti-HEV assay used in the study was produced with a recombinant peptide of the major HEV structural protein that occurs naturally as a homodimer (*36*). The 3-dimensional structure of the homodimer appears to model the HEV neutralization sites and other important HEV antigenic determinants located on the virus capsid (*34**,**37*). Consequently, IgG anti-HEV determined by this assay correlated with protective immunity (*27**,**38*). The antibody is regularly detected in acute- and convalescent-phase serum samples from hepatitis E patients and persists in primates for more than a year after HEV challenge (*32*). In our study, the average negative seroconversion rate of the antibody produced in response to asymptomatic HEV infection was 1.4% per year. This shows that the IgG antibody determined by this assay is stable and could provide a reliable epidemiologic marker to study HEV infection.

Phylogenetic analyses were conducted by using alignments of a 150-nt ORF2 sequence of 24 isolates from serologically confirmed hepatitis E cases. Based on previous studies (*6**,**33*), phylogenetic relationships established on the basis of partial sequences, such as this, are expected to be similar to relationships established from a complete sequence of the viral genome. The 24 virus strains analyzed from hepatitis E patients admitted to local hospitals during the time of the study are generally representative of the virus population prevalent in southern China where our study took place. All 24 isolates are genetically distinct and consist of 1 genotype 1 isolate and 23 genotype 4 isolates.

Zheng et al (*31*) studying HEV infection in 2 swine farming districts of eastern China showed that most HEV infections were genotype 4, which freely cocirculates among swine and humans. Since viral burden is much higher in swine than in humans, the authors concluded that swine were the principal source of genotype 4 virus for human infection. This contention is supported by seroepidemiologic findings, which suggested that infection may be acquired by contact with these animals or their sewage. Swine may be postulated to represent the principal reservoir of HEV in this current study area also because the prevalent virus population almost entirely comprises genotype 4 isolates and virtually all families raise pigs near their homes. According to this view, the level of infection among humans in each community would be determined primarily by the levels of infection of its swine population. Since the animals are kept close to family homes, humans could be the principal means by which the infection is spread among communities. However, the levels of infection differ substantially among communities, ranging from 25% to 66%, and a silent outbreak occurring in 1 of the communities during the time of the study involving 18% of its population did not spread to neighboring communities. This finding suggests that compared with swine, humans may not be an efficient vector for spreading genotype 4 virus infection.

The cross-sectional seroepidemiologic study showed that HEV infection is endemic in southern China. The average IgG anti-HEV seroprevalence of the population is 43%; participants' ages, sex, and communities are independent determinants. Seroprevalence was found to increase with age at a relatively constant rate of ≈1% per year until 59 years of age, and then remained essentially constant for older age groups. This finding suggests that HEV infection might have been endemic in southern China for > 60 years, so that persons in the 55- to 59-year age group may have been subjected to the same cumulative life-long exposure as older age groups. This estimate is consistent with findings from archival serum samples which indicate that HEV was prevalent in India before 1955 (*39*).

Our study identified 2 additional risk factors relating to the life style of the study participants. Seroprevalence was ≈2-fold higher for male participants than for female ones after 30 years of age but was similar for both sexes in the younger age groups. This finding probably reflects different roles adopted by males and females once families are established. Moreover, based on results from the follow-up study, the positive seroconversion rate is ≈3-fold higher among those 25–29 years of age than at other ages, which may be related to increased socialization during this stage of life.

In summary, our study showed that HEV infection is endemic in southern China and may have been so for at least 60 years. The prevalent virus is dominated by genetically diverse genotype 4 viruses. Among our study participants, infections seem to be mainly acquired from contact with swine; human-to-human transmission was of secondary importance. Herd immunity is built up separately to differing levels among different communities, leaving substantial proportions of the population vulnerable to HEV. Under such settings, vaccination programs for humans and swine could serve to boost herd immunity among humans and reduce the viral burden of swine herds.
